# Dialysis disequilibrium syndrome: an underdiagnosed condition? Results from a monocentric observational study

**DOI:** 10.1093/ckj/sfag171

**Published:** 2026-05-27

**Authors:** Théo Servan-Schreiber, Guillaume Lano, Matthieu Giot, Océane Jehel, Marion Pelletier, Marion Sallée, Philippe Brunet, Stéphane Burtey, Thomas Robert

**Affiliations:** Centre of Nephrology and Renal Transplantation, Hôpital de la Conception, CHU de Marseille, Marseille, France; Service de Néphrologie, Centre Hospitalier de Bastia, Bastia; Centre of Nephrology and Renal Transplantation, Hôpital de la Conception, CHU de Marseille, Marseille, France; Centre of Nephrology and Renal Transplantation, Hôpital de la Conception, CHU de Marseille, Marseille, France; Centre of Nephrology and Renal Transplantation, Hôpital de la Conception, CHU de Marseille, Marseille, France; Centre of Nephrology and Renal Transplantation, Hôpital de la Conception, CHU de Marseille, Marseille, France; C2VN, Aix Marseille Univ, INSERM, INRAE, Marseille, France; Centre of Nephrology and Renal Transplantation, Hôpital de la Conception, CHU de Marseille, Marseille, France; Centre of Nephrology and Renal Transplantation, Hôpital de la Conception, CHU de Marseille, Marseille, France; C2VN, Aix Marseille Univ, INSERM, INRAE, Marseille, France; C2VN, Aix Marseille Univ, INSERM, INRAE, Marseille, France; Service De Néphrologie et néphrogénomique, Hôpital Saint Joseph, Marseille, France

**Keywords:** chronic kidney disease, dialysis disequilibrium syndrome, fluid overload, intradialytic hypertension, metabolic acidosis

## Abstract

**Background:**

Dialysis disequilibrium syndrome (DDS) is a neurological complication occurring during hemodialysis initiation whose pathogenesis remains incompletely understood. Limited prospective adult data exists on DDS incidence and risk factors.

**Methods:**

We conducted a prospective observational study (February 2021–February 2022) including 48 patients initiating hemodialysis. DDS was assessed using a standardized severity score (0–3) before and after each of the first four sessions, defined as any increase in post-session score (**Δ Score ≥ 1**). Univariate and multivariate logistic regression identified risk factors.

**Results:**

Among 48 patients (70.8% male, median age 67 years), 22.9% developed DDS with two patients experiencing multiple episodes. Univariate analysis identified centrally acting agent use (45.5% vs. 8.1%, *P* = .01), fluid overload (45.4% vs. 13.5%, *P* = .03), lower pre-dialysis pH (7.28 vs. 7.37, *P* = .04), and higher chloride (100 vs. 94 mmol/l, *P* = .01) as significant associations. Pre-dialysis urea was not associated with DDS (*P* = .15). Multivariate analysis identified intradialytic hypertension as an independent risk factor (Odds ratio = 4.43, 95% confidence interval: 1.38–18.15, *P* = .01) occurring in 78.6% of DDS sessions versus 40.0% of non-DDS sessions.

**Conclusion:**

DDS is a common complication (22.9% incidence) with intradialytic hypertension as the key independent predictor. Pre-dialysis urea concentration was not predictive. These findings suggest a pathophysiological model involving acid-base dynamics and intracranial pressure regulation rather than solute gradients alone. Future multicenter studies are needed to validate these findings and optimize dialysis initiation strategies.

KEY LEARNING POINTS **What was known:**

- Dialysis disequilibrium syndrome (DDS) is a neurological complication of hemodialysis attributed to the “reverse urea effect” which has led to the standard practice of initiating hemodialysis with low-efficiency clearance. This urea-centric model has been challenged, suggesting other mechanisms. Incidence and the full spectrum of risk factors in adult cohorts remained undefined due to a lack of prospective studies.

**This study adds:**

- This first prospective study to determine DDS incidence in adults revealed a rate of 22.9%. Contrary to the “reverse urea effect” hypothesis, pre-dialysis urea levels were not associated with DDS. Instead, metabolic acidosis, fluid overload, and the use of centrally acting antihypertensive agents, emerged as significant contributing factors. Most importantly, intradialytic hypertension was identified as the sole independent predictor of DDS in multivariate analysis.

**Potential impact:**

- These findings suggest a shift toward a multifactorial approach for DDS prevention. Clinicians should consider screening for fluid overload and metabolic acidosis at dialysis initiation and recognize intradialytic hypertension as a critical clinical marker of evolving DDS. In high-risk patients, particularly those with severe acidosis (pH <7.20) or those developing IDH, preventive strategies such as using a lower dialysate bicarbonate concentration or shortening initial session durations may be warranted.

## INTRODUCTION

Dialysis disequilibrium syndrome (DDS) is a cluster of neurological symptoms that occur mostly during or shortly after the initiation of chronic hemodialysis (HD) in patients with chronic kidney disease [1]. Clinical presentations range from mild and self-limited manifestations—such as headache, nausea, vomiting—to severe, life-threatening neurological disturbances, including seizures, coma, and rarely death [2, 3]. The non-specific nature of DDS symptoms complicates diagnosis, which relies on a process of exclusion after ruling out other neurological etiologies. Despite its potential for significant morbidity and mortality, DDS remains underrecognized in clinical practice.

The classical understanding of DDS centers on the “reverse urea effect.” During rapid HD, small solutes such as urea are efficiently removed across the dialysis membrane, diffusing far more rapidly from the blood into the dialysate than from brain interstitium into blood. This creates an osmotic gradient favoring water movement into the brain interstitium and intracellular compartments, potentially leading to cerebral edema and neurological dysfunction, as confirmed by animal studies [4, 5]. The risk of DDS is enhanced with high-efficiency dialysis, emphasizing the need for cautious dialysis prescription adjustments to mitigate its occurrence particularly for HD initiation [6]. However, this model does not fully explain all clinical observations of DDS.

Alternative mechanisms have been proposed recently. Brain cells respond to chronic hyperosmolarity in uremia by accumulating organic osmolytes (amino acids, polyols, taurine) and idiogenic osmoles. These adaptations persist at initiation of HD, maintaining an osmotic gradient that drives water influx despite rapid urea removal [2, 7–9, 10]. Studies in uremic animals have shown that rapid correction of blood acidosis can paradoxically induce CSF acidosis and cerebral edema, potentially triggering DDS [9]. Various treatments, such as hypertonic solutions and cerebrospinal fluid (CSF) bypass techniques, have been explored to prevent or manage DDS, though their efficacy remains uncertain [3, 6, 10–13]. DDS is recognized as a complication of HD initiation, particularly in individuals with uremia. However, the evidence supporting these risk factors comes mainly from case reports and animal models, with limited prospective data in adult human cohorts.

To date, no large prospective observational study has systematically characterized DDS incidence, or comprehensively evaluated multiple potential risk factors in adult patients initiating HD. This study aimed to determine DDS annual incidence, identify associated risk factors, and evaluate the adequacy of traditional explanatory mechanisms.

## MATERIALS AND METHODS

### Patient population

We conducted a prospective observational study between February 2021 and February 2022 at the University Hospital La Conception, Marseille. The study was approved by the Research Ethics Committee and the Health Data Portal of Assistance Publique–Hôpitaux de Marseille (Registry No. PADS21-48). All participants received oral and written information, and verbal consent was obtained before enrollment.

We conducted a strictly prospective observational study. All patients initiating HD were screened and followed using predefined data collection forms. Inclusion criteria required complete clinical and biochemical data for all four initial sessions to ensure longitudinal integrity. Of the 84 patients screened, 36 were excluded: 28 due to missing data for at least one session, 5 due to pre-existing neurological conditions (seizure disorders, dementia, recent stroke, intracranial hemorrhage, or marked cerebral atrophy), 2 were aged under 18 years, and 1 provided incomplete informed consent.

### Dialysis initiation protocol

All patients followed a standardized, institution-specific dialysis initiation protocol (Supplementary Table S1). Dialysis efficiency was progressively increased across four sessions to minimize osmotic stress while achieving full adequacy by the fourth treatment. This protocol was used as our center standard of care at the time of the study. In order to evaluate our clinical practices, it seemed relevant to maintain this protocol.

Blood and dialysate flow rates were gradually escalated (QD 300→500 ml/min, QS 200→300 ml/min), along with dialyzer surface area (1.4→2.1 m²) and session duration (90→240 min). Dialysate potassium and calcium concentrations were individualized, whereas sodium (138 mmol/l) and bicarbonate (32 mmol/l) were standardized. Ultrafiltration targets were determined by clinical assessment of volume status. In cases of marked fluid overload, isolated ultrafiltration was performed at the start of dialysis. All patients were treated with high-flux HD. This relatively intensive initiation protocol, characterized by a rapid increase in dialyzer surface area and session duration, represented the established standard of care at our institution during the study period for patients requiring rapid uremic clearance. Continuous unfractionated heparin infusion was administered for sessions lasting >2 h, using weight-based dosing [14]. This protocol allowed gradual patient adaptation and minimized osmotic disequilibrium.

### DDS assessment

DDS symptoms were evaluated using a standardized form completed by blinded clinicians immediately before and within 30 minutes after each session. Symptom severity was graded on a three-stage scale:

- **Stage 1:** mild symptoms (nausea, vomiting, cramps, headache);
- **Stage 2:** moderate symptoms (visual disturbance, confusion, altered consciousness);
- **Stage 3:** severe manifestations (seizures, coma, death).

DDS was diagnosed when the post-session score exceeded the pre-session score by ≥1 (Δ Score ≥1). This sensitive definition captured mild-to-moderate cases often missed in routine practice. Severe DDS was defined as Δ Score ≥2 or any Stage 3 event. This approach differs from the original Port *et al*. criteria (requiring ≥3 mild, ≥2 moderate, or ≥1 severe symptom) and was adopted to improve clinical sensitivity and early detection.

### Definitions of clinical parameters

Emergency dialysis initiation was defined as an unplanned initiation in a patient hospitalized for acute complications of kidney failure, such as refractory fluid overload, severe uremia, or life-threatening electrolyte disturbances, requiring HD within 24–48 h of admission. Uremic syndrome was defined by the presence of at least two symptoms among nausea, vomiting, pruritus, encephalopathy, or significant weight loss (>5% of body weight in the preceding month). Uremic encephalopathy specifically required the presence of at least two neurologic signs, including confusion, altered consciousness, or disorientation, not explained by other clinical causes such as infection or medication, as documented by the attending physician prior to the dialysis session.

Metabolic indications for dialysis included refractory hyperkalemia, hyperphosphatemia, hypocalcemia, or metabolic acidosis not controlled by medical therapy.

Fluid overload was defined by clinical signs (peripheral edema, pulmonary crackles), radiographic evidence of congestion, or rapid weight gain (≥5% above dry weight).

Intradialytic hypertension (IDH) was defined as a rise in systolic blood pressure ≥20 mmHg and/or diastolic ≥10 mmHg during dialysis, with post-dialysis systolic >140 mmHg or diastolic >90 mmHg.

Intradialytic hypotension was defined as a systolic fall ≥20 mmHg, diastolic fall ≥10 mmHg, or absolute BP <90/60 mmHg during the session.

### Statistical analysis

Categorical variables were expressed as frequencies and percentages, while continuous variables were expressed as median and interquartile range (25th–75th percentile). Categorical variables were compared using the Chi-square test or Fisher’s exact test when applicable. Continuous variables were analyzed using the Student’s *t*-test or Mann-Whitney *U*-test, depending on data distribution. Univariate logistic regression was used to analyze DDS-associated risk factors, with results expressed as odds ratios (OR) and 95% confidence intervals (95% CI). Multivariate model was built by selecting independent variables based on a *P*-value less than .1 in univariate analysis and deemed clinically relevant. A significance threshold of 5% (*P* < .05) was applied. To investigate the association between antihypertensive drug classes and IDH, a per-session analysis was conducted on the study cohort (*n* = 187 evaluable sessions). A multivariable logistic regression model was constructed to identify independent predictors of IDH, adjusting for all antihypertensive classes [angiotensin-converting enzyme inhibitors (ACEi)/ angiotensin II receptor blockers (ARB), calcium channel blockers, diuretics, centrally acting agents, and beta-blockers], baseline hypertension status, total ultrafiltration volume, and session number. A post-hoc power analysis was performed for the primary association between IDH and DDS using a two-sided test of independent proportions (α = 0.05). Statistical analyses were conducted using IBM SPSS Statistics (version 20). Figures were generated using GraphPad Prism (GraphPad Software, San Diego, CA, USA) and JMP Pro (version 14).

## RESULTS

### Study population and baseline characteristics

**Figure 1:**
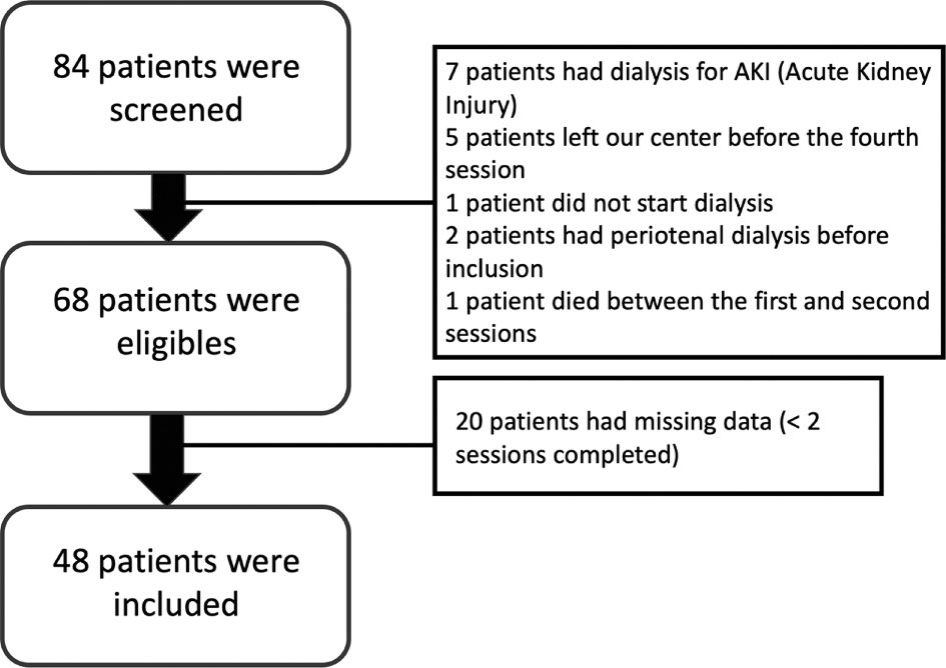
Flow chart.

During the 12-month study period, 84 patients initiated HD at our center. After a comprehensive review of clinical, biological and dialysis session data, cases with missing data were excluded resulting in a final cohort of 48 eligible patients (Fig. 1, Table 1 and 2). The characteristics of the 145 dialysis sessions analyzed are summarized in Table 3 and 4. The median age was 67 years old [48.5–78] with 70.8% male predominance. Most (56.2%) were overweight/obese. Hypertension (87.5%), diabetes (43.8%), undernutrition (22.9%), and heart failure (18.8%) were the most common comorbidities. Antihypertensive use included ACE inhibitors/ARBs (45.8%), calcium channel blockers (45.8%), diuretics (66.7%), beta-blockers (39.6%), and centrally acting agents (16.3%) . At dialysis initiation, 85.4% had eGFR <15 ml/min/1.73 m² (20.8% with graft failure). Most patients (75%) were hospitalized and 66.7% initiated dialysis emergently. Indications for initiation were uremic syndrome (50%), fluid overload (20.8%), and metabolic disorders (29.2%) (Table 1). Baseline biological characteristics showed median hemoglobin 9.6 g/dl [8.1–10.5], urea 42 mmol/l [35.4–51.8], and creatinine 717 µmol/l [440–990]. Most patients exhibited metabolic acidosis with a median pH 7.34 [IQR 7.27–7.39] and median bicarbonate 18.8 mmol/l [IQR 15.0–22.7] (Table 2).

**Table 1:** Comparative clinical baseline characteristics between DDS and control groups.

	Population (*n* = 48)	DDS− (*N* = 37)	DDS+ (*N* = 11)	*P*
**Age (years-old)**	67 [48.5–78]	68.5 [50–79]	64 [40–77]	.69
**BMI (%)**				
**<18**	2 (4.2)	2 (5.4)	0 (0.0)	.29
**18–25**	19 (39.5)	13 (35.1)	6 (54.5)	.29
**25–29**	19 (39.5)	14 (37.8)	5 (45.5)	.29
**>30**	8 (16.7)	8 (21.6)	0 (0)	.29
**Men (%)**	34 (70.8)	28 (75.7)	6 (54.5)	.26
**kidney transplantation failure (%)**	10 (20.8)	8(21.6)	2 (18.2)	1
**Nephrological follow-up (%)**	37 (77.1)	29 (78.4)	8 (72.7)	1
**Initiation of hemodialysis: (%)**				
-Emergency/programmed	32 (66.7)/16 (33.3)	27 (73)/10 [27]	5 (45.5)/6 (54.5)	.15
**Vascular access: (%)**				
- Catheter tunneled/transitory	22 (45.8)/9 (18.8)	17 (45.9)/7 (18.9)	5 (45.5)/2 (18.2)	1
-Arteriovenous fistula	17 (35.4)	13 (35.1)	4 (36.3)	
**Residual diuresis (%)**	47 (98)	37 (100)	10 (90.9)	.23
**Indication to start hemodialysis. (%)**				
-Uremic syndrome	24 (50)	21 (56.8)	3 (27.2)	**.07**
-Weight loss in the previous month	13 (27.1)	12 (32.4)	1 (9.1)	.14
-Uremic encephalopathy	17 (35.4)	15 (40.5)	2 (18.2)	.28
-Uremic pericarditis	1 (2.1)	1 (2.8)	0 (0)	1
-Pruritus	9 (18.8)	9 (24.3)	0 (0)	**.09**
-Nausea	23 (47.9)	18 (48.6)	5 (45.5)	1
-Vomiting	15 (31.3)	13 (35.1)	2 (18.2)	.46
-Fluid overload	10 (20.8)	5 (13.5)	5 (45.5)	**.03**
-Metabolic disorder	14 (29.2)	11 (29.7)	3 (27.2)	.47
**Comorbidities: (%)**				
Cirrhosis	1 [2]	1 (2.7)	0 (0.0)	1
Active cancer	4 (8.3)	3 (8.1)	1 (9.1)	1
Heart failure	9 (18.8)	8 (21.6)	1 (9.1)	.44
Arterial hypertension	42 (87.5)	32 (86.5)	10 (90.9)	1
Diabetes	21 (43.8)	19 (51.4)	2 (18.2)	**.08**
Undernutrition	11 (22.9)	10 [27]	1 (9.1)	.27
Dyslipidemia	5 (10.4)	3 (8.1)	2 (18.2)	.59
Smokers	19 (39.6)	14 (37.8)	5 (45.5)	.73
**Neurological comorbidity: (%)**	2 (4.2)	2 (5.4)	0 (0.0)	–
**Neurological treatment (%)**	11 (22.9)	9 (24.3)	2 (18.2)	1
**Antihypertensive drugs (%)**				
-ACEIs or ARBs	22 (45.8)	16 (43.2)	6 (54.5)	.74
-Calcic inhibitors	22 (45.8)	18 (48.6)	4 (36.4)	.75
-Diuretics	32 (66.7)	24 (64.9)	8 (72.7)	.73
-Centrally acting agents	8 (16.7)	3 (8.1)	5 (45.5)	**.01**
-Beta-blockers	19 (39.6)	15 (40.5)	4 (36.4)	1
Hospitalization (%)	36 (75)	28 (75.7)	8 (72.7)	1
Death at 3 months (%)	2 (4.1)	2 (5.4)	0 (0)	1
Time between two sessions (h)	48 [47.2–71.2]	48 [47.2–71.2]	48.1 [47.25–71.3]	.7

a Neurological comorbities: leukoaraiosis, hemorrhagic stroke, cerebral tumor, -subdural hematoma, intracerebral bleeding, cortical atrophia, microbleeds, cognitive trouble, dementia.

b **Neurological treatment:** Antidepressant/antiepilectic/neuroleptics/hypnotics/anxiolytics. **ACEis**, angiotensin-converting enzyme inhibitors **; ARA2:** angiotensin II receptor blockers (ARBs), **BMI, body mass index ; DDS–:** patients without dialysis disequilibrium syndrome during dialysis initiation ; **DDS+:** patients with dialysis disequilibrium syndrome during dialysis initiation.

**Table 2:** Comparative pre-dialysis biological characteristics at dialysis initiation.

	Population (*n* = 48)	DDS− (*n* = 37)	DDS + (*n* = 11)	*P*
Hemoglobin (g/dl)	9.6 [8.1–10.5]	9.6	9.05	.44
Urea (mmol/l)	42 [35.4–51.8]	43.1	39.8	.15
Creatinine (umol/l)	717 [440–990]	675	855	.76
Calculated Blood osmolarity (mmol/l)	318 [309–330.7]	318	319	.83
Sodium (mmol/l)	135 [133–139.7]	135	139	.13
Potassium (mmol/l)	4.5 [3.9–5.2]	4.45	4.9	.9
Chloride (mmol/l)	97 [93–102.5]	94	100	**.01**
Bicarbonate (mmol/l)	18.8 [15 -22.7]	19.5	16.5	**.08**
Albumin (g/l)	32 [28.2–35]	32.2	28.2	.80
Glycemia (g/l)	1.26 [1.02–1.65]	1.28	1.09	.20
pH	7.34 [7.27–7.39]	7.37	7.28	**.04**
pO_2_ (mmHg)	46 [39–98.5]	50	44	.86
pCO_2_ (mmHg)	37.5 [31.75–43.8]	38	36	.59

**DDS–:** patients without dialysis disequilibrium syndrome during dialysis initiation. **DDS+:** patients with dialysis disequilibrium syndrome during dialysis initiation.

### Dialysis disequilibrium syndrome incidence and patient risk factors

**Figure 2:**
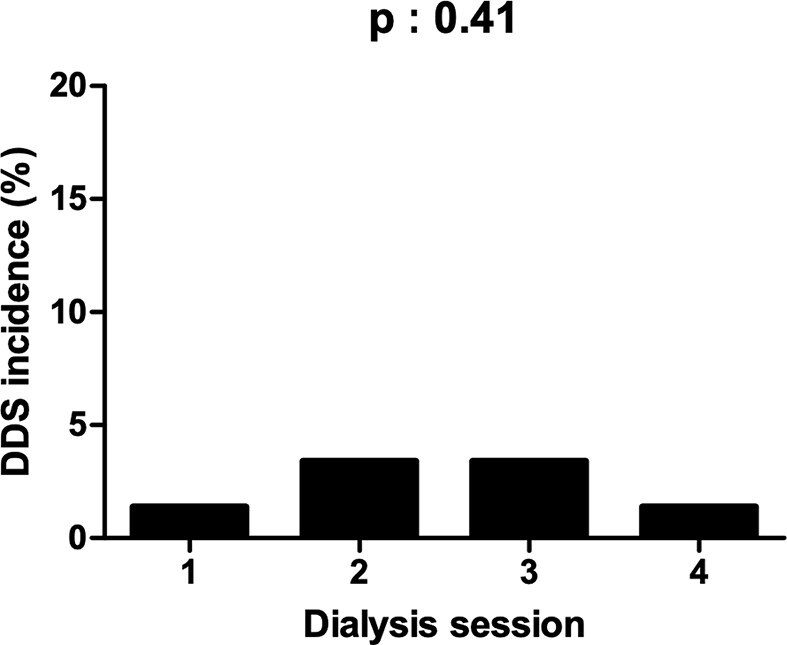
Incidence of DDS per session over the four sessions.

DDS was observed in 11 patients, representing an incidence of 22.9%. One patient developed severe DDS (2% of the cohort), and two patients manifested multiple DDS episodes, resulting in 14 DDS episodes out of 145 sessions (9.6%). Peak incidence occurred during the second and third sessions representing 71.4% of all DDS episodes (Fig. 2). Uremic symptom burden progressively declined, from 47.8% at baseline to 14.3% by the fourth session (Supplementary Fig. S1). 

**Figure 3:**
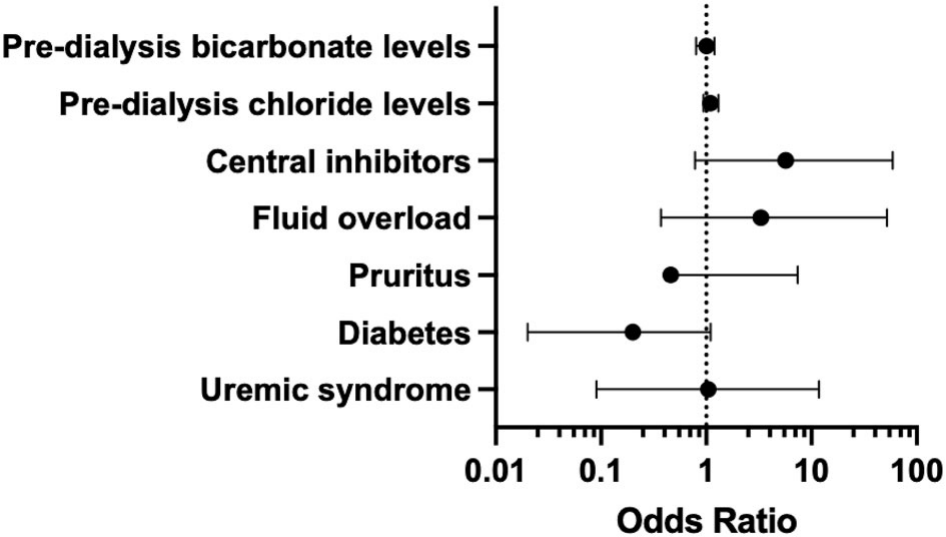
Forrest plot from multivariate analysis of pre-dialysis risk factors.

Among patients who developed DDS, there was a significantly higher prevalence of centrally acting agent use (45.5% vs. 8.1%, *P* = .01) and fluid overload (45.5% vs. 13.5%, *P* = .03) (Table 1). Pre-dialysis pH was significantly lower in the DDS group (7.28 vs. 7.37, *P* = .04), and pre-dialysis chloride levels were significantly higher (100 vs. 94 mmol/l, *P* = .01). Pre-dialysis urea concentration was not significantly associated with DDS development with DDS development (39.8 vs. 43.1 mmol/l, *P* = .15) (Table 2). Uremic syndrome, while more prevalent in non-DDS patients (56.8% vs. 27.2%), showed only a trend toward association (*P* = .07) (Table 1). Multivariate analysis of patient baseline characteristics did not identify any independent risk factors, although diabetes showed a trend toward a protective effect (Fig. 3) (Table 3). Mortality at 3 months was 4.2% (two deaths in the overall cohort), with no statistically significant difference between DDS and non-DDS groups (5.4% vs. 0%, *P* = 1.00). Neither deaths were attributable to DDS.

**Table 3:** Multivariate analysis of patients characteristics.

	OR [IC 95%]	*P*
Uremic syndrome	1.04 [0.09; 11.8]	.98
Diabetes	0.2 [0.02; 1.1]	.06
Pruritus	0.46 [0; 7.4]	.62
Fluid overload	3.3 [0.37; 52.3]	.28
Central inhibitor	5.7 [0.78; 59.3]	.09
Pre-dialysis chloride levels	1.09 [0.94; 1.3]	.26
Pre-dialysis bicarbonate level	1 [0.8; 1.2]	.97

Multivariate model built on independent variables based on a *P*-value < .1 in univariate analysis and clinical relevance.

### Intradialytic variables and DDS association

Across the 145 sessions, IDH occurred in 78.6% of DDS sessions compared to 40.0% of non-DDS sessions (*P* = .01) (Table 4, 5 and 3). In contrast, intradialytic hypotension did not differentiate the two groups (0% vs. 10.7%, *P* = .36). The median Kt/V across all sessions was 0.83 0.49–1.22. Although not statistically significant, DDS sessions showed numerically higher adequacy compared to non-DDS sessions (1.17 0.82–1.39 vs. 0.74 0.46–1.15, *P* = .15). Isolated ultrafiltration was performed in 7.5% (11/145) of sessions, with no significant difference between the two groups (*P* = 1.00).

**Table 4:** Comparative characteristics of dialysis sessions.

	Population (*n* = 145)	DDS−(*n* = 131)	DDS + (*n* = 14)	*P*
Weight (kg)	74 [64.7–80.5]	74 [64–81]	73.5 [57–75.5]	.08
Ultrafiltration volume (ml)	250 [200–250]	0 [0–225]	0 [0–1042]	.56
Max blood flow (ml/min)	141 [126–250]	250 [200–250]	250 [250–262]	**.08**
Kt/V	0.83 [0.49–1.22]	0.74 [0.46–1.15]	1.17 [0.82–1.39]	.15
Isolated ultrafiltration (%)	11 (5.8)	10 (7.7)	1 (7.1)	1
Duration of ultrafiltration only (min)	120 [60–150]	105 [60–127.5]	150 [150–150]	.23
Intradialytic hypotension (%)	14 (9.6)	14 (10.7)	0 (0)	.36
Intradialytic hypertension (%)	63 (43.4)	52 (40.0)	11 (78.6)	**.01**
Loss of dialysis circuit (%)	5 (3.4)	5 (3.8)	0 (0)	1
Early restitution (%)	2 (1.4)	1 (0.8)	1 (7.1)	.19
Na (mmol/l)	138 [138–138]	138 [138–138]	138[138–138]	.40
K (mmol/l)	2 [2–3]	2 [2–3]	2 [2–3]	.79
Ca (mmol/l)	1.75 [1.5–1.75]	1.75 [1.5–1.75]	1.625 [1.5–1.75]	.71
HCO_3_^−^ (mmol/l)	32 [32–32]	32 [32–32]	32 [31.5–32]	.36

Abbreviation: Kt/V represent dialysis adequacy parameter.

**Table 5:** Comparative pre-dialysis biological characteristics across the four sessions.

	Population (*n* = 145)	DDS– (*n* = 131)	DDS+ (*n* = 14)	*P*
Hemoglobin (g/dl)	9.1 [7.9–10.4]	9.1 [7.9–10.5]	8.8 [7.6–10.6]	.74
Hematocrite (%)	27 [24–32]	27 [24–32]	27 [24–31.5]	.84
Urea (mmol/l)	32.71 [25.34–40.66]	33.8 [26.9–42.9]	28 [21.8–39.9]	.05
Creatinin (umol/l)	578.9 [402.7–879.6]	566 [402–888]	677 [434–948]	.92
Calculated blood osmolarity (mmol/l)	310.2 [302.8–320]	312 [304–322]	308.5 [302.6–317.8]	.17
Sodium (mmol/l)	135 [132–138]	135 [132–139]	138 [134–140]	.30
Potassium (mmol/l)	4.21 [3.66–4.90]	4.2 [3.7–4.9]	3.6 [3.2–4.2]	**.02**
Chloride (mmol/l)	98 [94–101]	98 [93.3–101]	99 [96–103.5]	.17
Bicarbonate (mmol/l)	21.1 [17.8–23.4]	21.1 [17.9–23.8]	21.2 [16.1–24]	.96
Albumin (g/l)	30.9 [27–34.5]	30.5 [26.9–34.5]	28.2 [25.4–31.6]	.15
Glycemia (g/l)	1.36 [1.06–1.75]	1.35 [1.05–1.76]	1.23 [1.13–1.48]	.62
pH	7.37 [7.32–7.40]	7.37 [7.33–7.42]	7.37 [7.28–7.40]	.67
PO_2_ (mmHg)	49 [39–97]	49 [37.2–99.2]	45 [44–68.5]	.55
PCO_2_ (mmHg)	39 [34–43]	39 [33–44]	37 [32.75–43.5]	.90

**DDS–:** patients without dialysis disequilibrium syndrome during dialysis initiation. **DDS+:** patients with dialysis disequilibrium syndrome during dialysis initiation.

In a per-session analysis of the 145 sessions, the use of centrally acting antihypertensive agents was associated with a significantly higher occurrence of IDH (70.8% vs. 38.1%, *P* = .004). Multivariable logistic regression adjusting for all antihypertensive classes and ultrafiltration volume confirmed that centrally acting agents were the strongest independent predictor of IDH [adjusted odds ratio (aOR) 3.42; 95% CI 1.45–8.10; *P* = .004], while beta-blocker therapy was independently associated with a lower risk of IDH (aOR 0.38; 95% CI 0.15–0.92; *P* = .032) (Table 6 and 7). The post-hoc power analysis for the primary association between IDH and DDS yielded a power of 79.03%, confirming that the study was sufficiently powered to detect this association despite the sample size.

**Table 6.** Prevalence of intradialytic hypertension (IDH) by antihypertensive drug class (*n* = 145 sessions).

Drug class	Sessions exposed (*n*)	IDH Yes, *n* (%)	Prevalence of IDH
Centrally acting agents	24	17 (70.8)	70.8%
Beta-blockers	58	18 (31.0)	31.0%
Calcium channel blockers	66	36 (54.5)	54.5%
ACEi/ARB	68	32 (47.1)	47.1%
Diuretics	98	44 (44.9)	44.9%

Data are presented as the number and percentage of sessions. The analysis was conducted on 187 evaluable dialysis sessions from the study cohort (*n* = 48 patients). IDH, intradialytic hypertension; ACEi, angiotensin-converting enzyme inhibitor; ARB, angiotensin receptor blocker.

**Table 7:** Multivariable logistic regression analysis for predictors of intradialytic hypertension.

Predictor	Adjusted OR	95% CI	*P*-value
Centrally acting agents	3.42	1.45–8.10	.004
Beta-blockers	0.38	0.15–0.92	.032
Calcium channel blockers	1.88	0.88–4.02	.105
ACEi/ARB	1.42	0.62–3.25	.395
Diuretics	1.15	0.50–2.65	.740
Baseline hypertension (Yes)	0.62	0.20–1.85	.380
Total ultrafiltration (per 1 l)	1.00	0.70–1.42	.995

The model includes all listed antihypertensive drug classes entered simultaneously as binary indicators, along with baseline hypertension status (yes/no), total ultrafiltration volume (continuous, per 1 l), and session number (categorical, with Session 1 as reference). Odds ratios (OR) are adjusted for all other variables in the model. aOR, adjusted odds ratio; CI, confidence interval; ACEi, angiotensin-converting enzyme inhibitor; ARB, angiotensin receptor blocker; UF, ultrafiltration; HTA, hypertension.

### Multivariate analysis of DDS predictors

**Figure 4:**
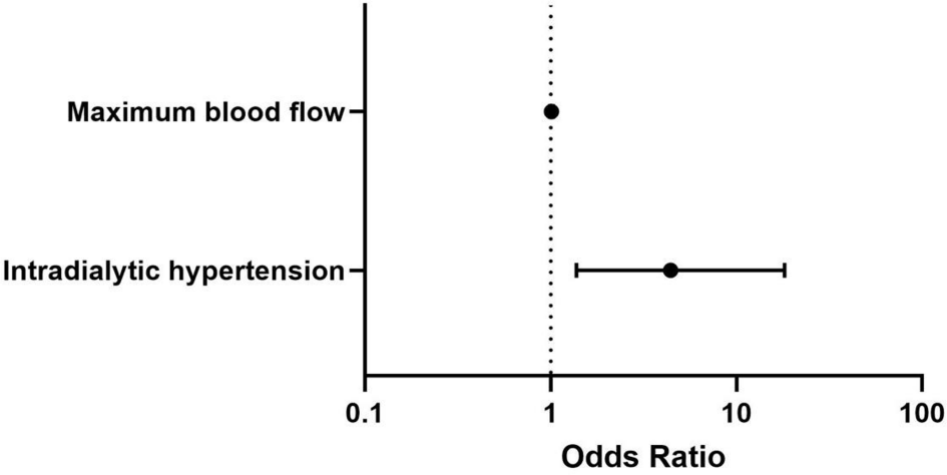
Forrest plot from multivariate analysis of intradialytic events associated with DDS.

Multivariate analysis of dialysis session-specific variables revealed that IDH was the only independent predictor of DDS (OR = 4.43, 95% CI: 1.38–18.15, *P* = .01) (Table 8 7). Maximum blood flow rate was not an independent predictor (*P* = .14). A post-hoc power analysis for the primary IDH-DDS association yielded a power of 79.03%, confirming that the study was sufficiently powered to detect this association despite the sample size. (Fig. 4, Table 8).

**Table 8:** Multivariate analysis of dialysis session characteristics.

	OR [IC 95%]	*P*
Intradialytic hypertension	4.43 [1.38; 18.15]	**.01**
Maximum blood flow	1.01 [0.99; 1.03]	.14

Multivariate model built on intradialytics variables based on a *P*-value < .1 in univariate analysis.

## DISCUSSION

This prospective observational study is the first to assess the incidence and risk factors of dialysis DDS in an adult cohort. We observed a notably high incidence of 22.9% which substantially exceeds the 4% reported in a recent pediatric study, although the rate of severe DDS (Δ Score ≥2 or Stage 3 symptoms) remained comparable [15]. This difference likely reflects our more sensitive diagnostic criteria, based on a differential score (Δ Score ≥1) designed to capture clinically relevant mild-to-moderate cases often overlooked in routine practice [3]. The peak incidence during the second and third sessions suggests that cumulative metabolic changes and patient adaptation over successive treatments play a critical role. Furthermore, the lack of significant difference in incidence across sessions indicates that patient-specific vulnerability, rather than session-related variables alone, predominantly influences DDS risk, necessitating vigilant monitoring throughout the initiation period.

Fluid overload emerged as a potential risk factor for DDS, representing the first such association demonstrated prospectively. We propose that in patients with marked volume expansion, a degree of subclinical interstitial or intracellular cerebral edema may already exist prior to dialysis. Rapid osmotic shifts during HD could exacerbate this pre-existing edema as water moves into brain cells along new osmotic gradients, a process possibly likely amplified by the 65% increase in aquaporin-4 expression and 50% reduced urea transporter expression in uremic brain tissue [16, 17]. Confirmation of this hypothesis requires further studies incorporating neuroimaging and intracranial pressure monitoring.

Our findings challenge the traditional “reverse urea effect” as the dominant mechanism of DDS [2 4, 6]. Neither pre-dialysis uremic syndrome nor urea concentration predicted DDS risk in our cohort. This supports a more nuanced model where DDS pathophysiology involves synergistic processes beyond simple small-solute shifts, potentially related to the persistence of idiogenic osmoles in the brain during rapid clearance.

Metabolic acidosis also correlated with DDS in univariate analysis, though not as an independent risk factor. These findings align with evidence implicating paradoxical cerebrospinal fluid acidosis in DDS pathogenesis. Athavale *et al*. reported a severe DDS case in a patient with profound metabolic acidosis and a minimal urea delta, suggesting the potential role of acid-base disturbances [18]. Studies in uremic dogs have shown a significant increase in intracranial pressure following HD, using higher bicarbonate dialysate concentrations (28 vs. 20 mmol/l) [19]. Similarly, in children with diabetic ketoacidosis treated with bicarbonate therapy in the setting of low pCO_2_ and high blood urea nitrogen, cerebral edema incidence increased substantially [9, 20]. These observations suggest that rapid systemic bicarbonate correction increases CO₂ levels, which diffuses across the blood-brain barrier faster than bicarbonate, inducing intracellular acidosis. This process activates Na⁺/H⁺ and Cl^−^/HCO₃^−^ exchangers, driving intracellular sodium and chloride accumulation and subsequent osmotic water influx.

The most striking finding was the independent association between IDH and DDS. Systemic and intracranial pressures are closely linked via cerebral autoregulation. Whether IDH represents a primary driver of cerebral edema or a secondary compensatory response to rising intracranial pressure—consistent with the Lund Concept—remains to be fully elucidated [28]. However, our per-session analysis revealed a critical link between antihypertensive management and IDH. Centrally acting agents were the strongest independent predictors of IDH (aOR 3.42, *P* = .004), possibly by suppressing dopamine-mediated inhibition of Na⁺/H⁺ exchange. Conversely, beta-blocker therapy was independently protective against IDH (aOR 0.38, *P* = .032), suggesting that stabilizing the sympathetic response may reduce the risk of cerebral hemodynamic disturbances.

Our novel finding of an association between centrally acting agents and IDH (aOR 3.25) further supports the role of the autonomic nervous system in DDS. Conversely, the independent protective effect of beta-blockers against IDH (aOR 0.42) suggests that stabilizing the sympathetic response during dialysis might mitigate the risk of cerebral hemodynamic disturbances. These results align with the Lund Concept, which emphasizes the physiological interconnection between systemic arterial pressure and intracranial pressure regulation.

Based on these findings, we propose modified strategies for patients at elevated risk:

1. **Screen** for fluid overload, metabolic acidosis (pH < 7.30), and central inhibitor use at dialysis initiation.
2. **Monitor** blood pressure closely during early sessions for signs of IDH.
3. **Consider** shortened initial sessions (120–150 min) in patients exhibiting IDH.
4. **Use** lower dialysate bicarbonate concentrations (24–28 mmol/l) for markedly acidotic patients (pH < 7.20). These pragmatic measures may reduce DDS incidence. An integrated model linking fluid overload, acidosis correction, and neuropharmacologic factors to cerebral edema and IDH is illustrated in Supplementary Fig. S1.

Our study has several limitations. The modest sample size (*n* = 48) restricted statistical power for patient-level multivariate analyses, although post-hoc analysis confirmed 79% power for the primary session-level association. The single-center design and the use of an intensive initiation protocol may limit the generalizability of our findings to centers with slower initiation strategies. Additionally, the absence of a systematic neuro-imaging prevented direct verification of cerebral edema and uremic toxins were not measured [23 27]. Nevertheless, the use of a standardized differential score and blinded clinical assessment significantly reduced diagnostic bias. Prior studies demonstrate close agreement between venous and arterial pH, suggesting minimal impact from our predominantly venous sampling [26].

## CONCLUSION

DDS is a frequently overlooked complication occurring at the onset of HD. Our study challenge the urea-centric paradigm, identifying metabolic acidosis, fluid overload, and IDH as the key clinical drivers. Intradialytic hypertension, strongly associated with the use of centrally acting agents and mitigated by beta-blockers, serves as a critical independent predictor and clinical signal of DDS. These observations support a more nuanced pathophysiological model and suggest that monitoring blood pressure during early dialysis sessions may identify patients at DDS risk. Multicenter prospective studies are warranted to validate these findings.

## Supplementary Material

sfag171_Supplemental_File
