# Supplementary material for: Dialysis disequilibrium syndrome: an underdiagnosed condition? Results from a monocentric observational study
Source: Clin Kidney J. 2026 May 27;19(7):sfag171. doi: 10.1093/ckj/sfag171 (PMC13320231; doi:10.1093/ckj/sfag171)
Supplement: sfag171_Supplemental_File [file sfag171_supplemental_file.pdf]

SUPPLEMENTARY MATERIAL

Supplementary Table 1 : Dialysis protocol for the first four sessions.

	Duration (min)	QD (ml/min)	QS (ml/min)	Dialyzer surface (m^{2})
Session 1	**90**	**300**	**200**	**1.4**
Session 2	**120**	**300**	**250**	**1.4**
Session 3	**180**	**400**	**250**	**2.1**
Session 4	**240**	**500**	**300**	**2.1**

QD: blood flow rate; QS: dialysate flow rate.

Supplementary Table 2: Dialysis Disequilibrium Syndrome (DDS) Scoring System: Symptom severity was graded based on the highest stage reached. DDS was defined as an increase in the post-session score compared to the pre-session score (Δ Score ≥ 1).

Stage severity	Symptoms
Mild (Stage 1)	Nausea/vomiting
	Muscles cramps
	Headache
Moderate (Stage 2)	Visual disturbance
	Confusion
	Abnormal movements
Severe (Stage 3)	Seizure
	Coma
	Death

Supplementary Table 3: Association between centrally acting antihypertensive agents and intradialytic hypertension (per-session analysis)

Centrally acting agents	No IDH, n (%)	IDH, n (%)	Odds Ratio (95% CI)	P-value
No (n = 121 sessions)	74 (61.2)	47 (38.8)	Reference	—
Yes (n = 24 sessions)	7 (29.2)	17 (70.8)	3.82 (1.48–9.85)	0.004

IDH: intradialytic hypertension.

Supplementary Figure 1 : Uremic Score Evolution Prior to Each Hemodialysis Session

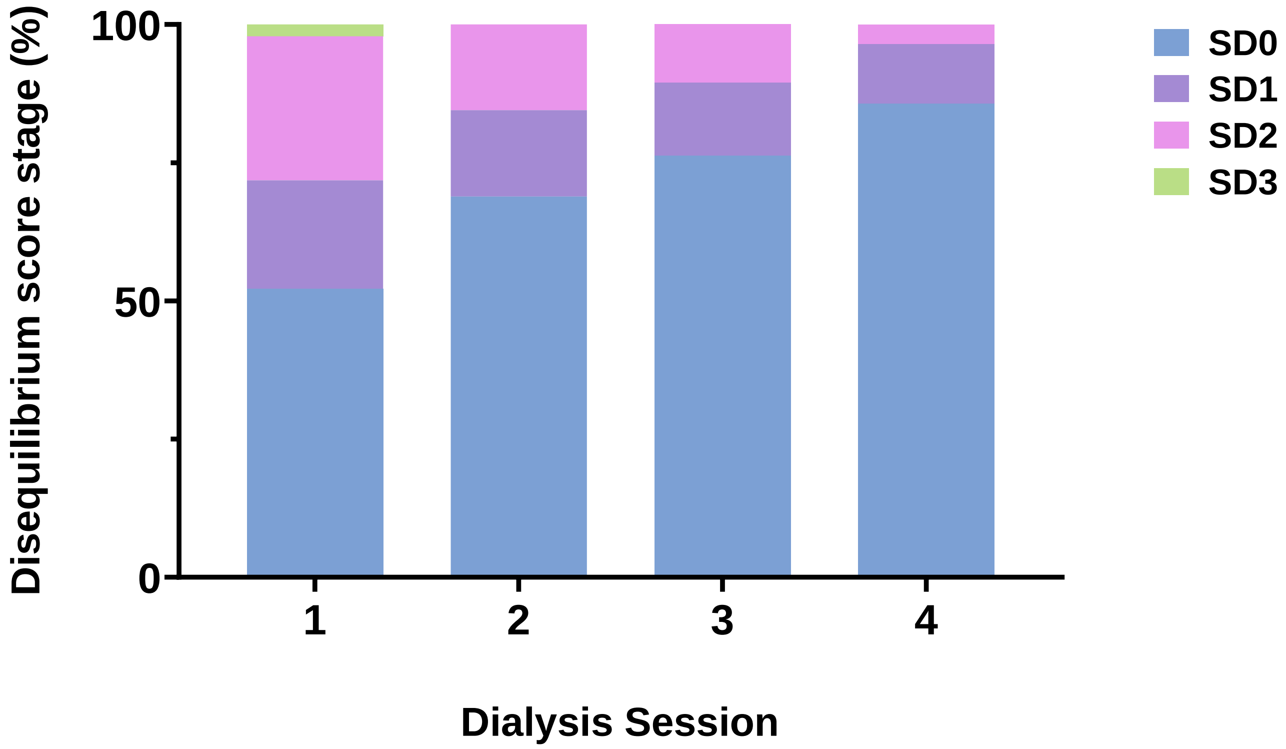

Since several symptoms are common to both uremic syndrome and dialysis disequilibrium syndrome, we tracked the evolution of pre-dialysis uremic symptoms throughout the follow-up using the standardized score. At baseline, 47.8% of patients expressed uremic symptoms prior to the first hemodialysis session. This proportion declined to 14.3% by the beginning of the fourth session, indicating progressive resolution of uremia. This methodology allowed for the clear distinction between pre-existing uremic manifestations and newly emergent DDS symptoms (defined as an increase in score during or after the session).

Supplementary Figure 2:

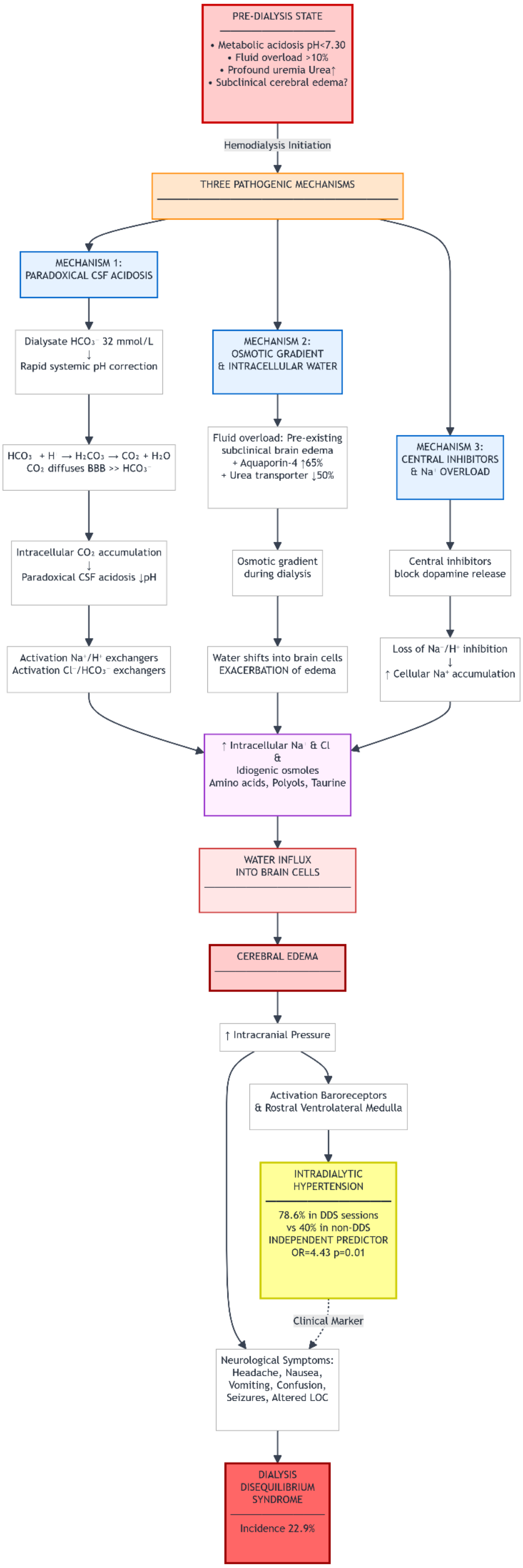

Supplementary Figure 2 legend: the proposed integrated pathophysiological model of DDS. Three distinct but interconnected mechanisms converge to produce cerebral edema: (1) paradoxical cerebrospinal fluid acidosis resulting from rapid CO_2 diffusion across the blood-brain barrier despite systemic pH correction by bicarbonate-based dialysate; (2) exacerbation of pre-existing subclinical brain edema in fluid-overloaded patients through osmotic water shifts amplified by increased aquaporin-4 expression (65% increase) and reduced urea transporter expression (50% reduction) in uremic brain tissue; and (3) cellular sodium accumulation driven by central inhibitor-mediated suppression of dopamine signaling and loss of normal Na^+/H^+ inhibition. These three mechanisms collectively activate intracellular ion exchangers (Na^+/H^+ and Cl^-/HCO_3^-), increase intracellular electrolyte concentration, accumulate idiogenic osmoles (amino acids, polyols, taurine), and promote osmotically driven water influx. The resulting cerebral edema elevates intracranial pressure, triggering compensatory intradialytic hypertension through baroreceptor activation and rostral ventrolateral medulla stimulation. Intradialytic hypertension thus serves as a clinical marker of this underlying hemodynamic cascade and may warrant intervention to prevent overt DDS manifestation.
